# Cytokine and chemokine alterations in tissue, CSF, and plasma in early presymptomatic phase of experimental allergic encephalomyelitis (EAE), in a rat model of multiple sclerosis

**DOI:** 10.1186/s12974-016-0757-6

**Published:** 2016-11-15

**Authors:** Nozha Borjini, Mercedes Fernández, Luciana Giardino, Laura Calzà

**Affiliations:** 1 Research and Development, Chiesi Farmaceutici S.p.A, Via Palermo 26/A, Parma, 43100 Italy; 2 Health Science and Technologies Interdepartmental Center for Industrial Research (HST-ICIR), University of Bologna, Via Tolara di Sopra 41/E, Bologna, Ozzano Emilia I 40064 Italy; 3 IRET Foundation, Via Tolara di Sopra 41/E, Bologna, Ozzano Emilia 40064 Italy; 4 Department of Veterinary Medical Sciences, University of Bologna, Via Tolara di Sopra 50, Ozzano Emilia, BO 40064 Italy; 5 Department of Pharmacy and Biotechnology, University of Bologna, Via Tolara di Sopra 41/E, Bologna, Ozzano Emilia 40064 Italy

**Keywords:** Neuroinflammation, Multiple sclerosis, Experimental allergic encephalomyelitis, Biomarkers, Plasma, CSF1

## Abstract

**Background:**

Experimental allergic encephalomyelitis (EAE) is the most commonly used experimental animal model for human multiple sclerosis (MS) that has been used so far to study the acute and remission-relapsing phases of the disease. Despite the vast literature on neuroinflammation onset and progression in EAE, important questions are still open regarding in particular the early asymptomatic phase between immunization and clinical onset.

**Methods:**

In this study, we performed a time-course investigation of neuroinflammation and demyelination biomarkers in the spinal cord (SC), cerebrospinal fluid (CSF), and blood in EAE induced in dark agouti (DA) female rats compared to the controls and adjuvant-injected rats, using high-throughput technologies for gene expression and protein assays and focusing on the time-course between immunization, clinical onset (1, 5, 8 days post-immunization (DPI)), and progression (11 and 18 DPI). The expression profile of 84 genes related to T cell activation/signaling, adaptive immunity, cytokine/chemokine inflammation, demyelination, and cellular stress were analyzed in the tissue; 24 cytokines were measured in the CSF and plasma.

**Results:**

The macrophage colony-stimulating factor (CSF1) was the first up-regulated protein as far as 1 DPI, not only in blood but also in CSF and SC. A treatment with GW2580, a selective CSF1R inhibitor, slowed the disease progression, significantly reduced the severity, and prevented the relapse phase. Moreover, both pro-inflammatory (IL-1β, TNF-α) and anti-inflammatory cytokines (IL-5, IL-10, VEGF) were up-regulated starting from 8 DPI. Myelin genes were down-regulated starting from 8 DPI, especially MAL, MBP, and PMP22 while an opposite expression profile was observed for inflammation-related genes, such as CXCL11 and CXCL10.

**Conclusions:**

This early cytokine and chemokine regulation indicates that novel biomarkers and therapeutic options could be explored in the asymptomatic phase of EAE. Overall, our findings provide clear evidence that CSF1R signaling regulates inflammation in EAE, supporting therapeutic targeting of CSF1R in MS.

## Background

Inflammation and demyelination are the primary pathological events in multiple sclerosis (MS), the most diffuse inflammatory demyelinating disease of the central nervous system (CNS) [1]. The progressive failure of remyelination leads to the cumulative loss of axons, gray matter atrophy, and the prevalent neurodegeneration responsible for chronic disability and cognitive defects [2]. Despite the intensive efforts and the major progress achieved in understanding the inflammatory process and pathogenetic mechanisms within this heterogeneous disease entity to date, the exact pathophysiological process of MS remains elusive.

Both humoral and the cell-mediated immune reactions having peripheral macrophages and central microglia as key players take part in the neural-immune mechanisms underlying MS [3]. Indeed, MS pathogenesis has been believed to be derived from autoreactive lymphocytes (T/B cells) and a plethora of macrophages recruited from the periphery. Both cell classes are able to cross the blood–brain barrier (BBB) [4] and to engage microglia, the resident immune cells in the CNS [5], ultimately leading to severe demyelination and neuronal degeneration. Cytokines and chemokines play a key role in these processes by regulating cell migration, proliferation, and activation of resident and infiltrating cells [6].

An important contribution to MS studies has been made by experimental allergic encephalomyelitis (EAE), the most widely used animal model for MS [7], in which the interaction between a variety of immunopathological and neuropathological mechanisms affords an approximation of the key pathological features of MS pathology, including inflammation and immune reaction, demyelination, axonal loss, and gliosis [8, 9]. EAE has been also used to develop and validate all approved therapies for MS [10], thus confirming the good correlation between animal and human pathology [11, 12].

In particular, the EAE model allows a hitherto poorly studied phase of the disease to be investigated, i.e., the preclinical asymptomatic phase. This time window could be particularly interesting for the discovery of novel early biomarkers and novel therapeutic target. According to recent reports, in the MS murine model, a significant T cell mobilization was observed at 2 days post-immunization (DPI), thus an asymptomatic stage of the disease [13]. Furthermore, a transient vessel leak in the cortical gray matter has been described at the same time window [14]. Moreover, pain associated with microglia and astrocyte activation occurs in EAE prior to the onset of clinical signs [15, 16].

It is worthy of note that few studies to date have been devoted to the investigation of this asymptomatic phase of the disease. In order to elucidate very early molecular alterations occurring in the CNS of EAE animals before the clinical onset of the disease and to link tissue alterations to potential CSF and plasma biomarkers, in the present study, we investigated molecular mediators of neuroinflammation and demyelination in the tissue (SC), CSF, and plasma during early presymptomatic EAE using high-throughput technologies for gene expression and protein assays. The results indicate that the profile of neuroinflammation and demyelination biomarkers is dramatically changed during the early phase of EAE. Consequently, we have identified the regulation of the chemokine macrophage colony-stimulating factor (CSF1) already 24 h after immunization, which indicates the occurrence of microglia activation. Interestingly, we found that a selective inhibition of CSF1R significantly reduced the severity of the disease and prevented the relapse phase in EAE rats, suggesting the importance of CSF1-CSF1R signaling in microgliosis and inflammation in MS.

## Methods

### Animals, EAE induction, and disease follow-up

Dark agouti (DA) (Harlan, Italy) female rats, 151–167-g body weight, were used in the first part of the study, placed on ad libitum food and water, and housed three per cage on a 12-h light/dark cycle. The DA rat model of EAE mimics certain aspects of the clinical course of the disease in people with RRMS [17–19], typified by progressive, sustained demyelination, and associated axonal loss [20, 21]. The disease onset in this strain bears is characterized by neurological impairments, manifested as a flaccid tail followed by an acute attack with disturbed gait and paralysis. Most DA animals recover spontaneously from paralysis and experience remission and may then undergo one or more relapses.

A group of 42 rats was sensitized (considering group composition and two extra animals) by a medium containing 0.15 mg/ml guinea pig spinal cord tissue in complete Freund’s adjuvant (CFA, Sigma, Saint Louse, USA), 50% *v*/*v*, to which 5 mg/ml of heat-inactivated *Mycobacterium tuberculosis* (Difco H37Ra, DB, Milan, Italy) was added. Sensitization was performed by injecting 100 μl in both hind pads. Control rats (*n* = 8) and adjuvant-injected rats (CFA, 50% *v*/*v*, heat-inactivated *M. tuberculosis*, 5 mg/ml) (*n* = 15) were used. The rats were weighed daily and examined for clinical signs of EAE, according to the following semi quantitative score for neurological disability: 0 = no signs, 1 = loss of tail tone, 2 = mono or bilateral weakness of hind legs or middle ataxia, 3 = ataxia or paralysis, 4 = severe hind legs paralysis, and 5 = severe hind leg paralysis and urinary incontinence [22]. In view of the animals’ disability, wet food was included inside the cages to facilitate feeding. At 1, 5, 8, 11, and 18 DPI, eight EAE rats were randomly sacrificed. From each experimental group, five animals were used for proteomic and molecular biology experiments and three for morphology and immunohistochemistry. All animal protocols described herein were carried out in accordance with the European Community Council Directives (86/609/EEC), approved by the intramural ethical committee for animal experimentation of Bologna University and the Ministry of Health (no. 158/2013-B) and comply with the guidelines published in the *NIH Guide for the Care and Use of Laboratory Animals* [23].

### GW2580 treatment and CSF1R inhibition

In order to calculate the number of animals needed to study the effect of the treatment with GW2580, we performed a power analysis using the G*Power 3.1 software. To reach a power of 0.9, based on retrospective analysis of recent research done by others [24, 25], we needed a minimum of *n* = 6 animals per experimental group.

The experiments were designed in compliance with the ARRIVE guidelines, randomizing the procedures and applying blinded analysis.

GW2580 (LC Laboratories, Boston, USA) was dissolved in 0.5% hydroxypropyl methylcellulose and 0.1% Tween 80 [26, 27]. The experimental groups made of *n* = 6 animals each were as follows: vehicle (0.5% hydroxypropyl methylcellulose/0.1% Tween 80); control + GW2580; EAE; and EAE + GW2580. The rats (control + GW2580 and EAE + GW2580 groups) were under GW2580 treatment at 40 mg/kg once daily by oral gavage using flexible plastic feeding tubes FTP-15-78-50 (Instech Laboratories, Netherlands) for 1 day prior and 11 consecutive days following the immunization. All animals were weighed daily and the EAE and EAE + GW2580 groups were examined for clinical signs of EAE till the 18 DPI (last day of the experiment) following the semi quantitative score for neurological disability as already explained in the first part of the study. Animal protocols described herein were carried out in accordance with the European Community Council Directives (86/609/EEC), approved by the intramural ethical committee for animal experimentation of Bologna University and the Ministry of Health (no. 607/2016-PR).

### Histology and immunohistochemistry

On post-immunization days 1, 5, 8, 11, and 18, three rats were scarified and the lumbar spinal cord (LSC) tissues of the control, EAE, and adjuvant groups were fixed in 4% paraformaldehyde and picric acid and saturated aqueous solution in 0.1 M Sörensen buffer pH 7 for 24 h and then washed with 5% sucrose solution every day till the fixative was completely removed. The tissues were frozen with CO_2_ gas and kept at −80 °C until processed. Coronal sections (14-μm thickness) were then prepared (Microm HN550, Bio-Optica, Milan, Italy) and processed for morphological studies. For immunohistochemistry, the sections were first rehydrated and then incubated for 1 h with PBS–0.3% Triton X-100, 2% normal serum goat, and 1% BSA, followed by incubation with the primary antibodies diluted in the pre-absorption solution overnight at 4°C. The primary antibodies and dilutions used were as follows: rabbit anti-CD44 (1:100, Cluster of differentiation 44, Acris Antibodies, Inc, San Diego, USA), rabbit anti-CD163 (1:100, Cluster of differentiation 163, Bioss Inc, Woburn, USA), mouse anti-CD86 (1:250, Cluster of differentiation 86, Novus Biological Europe, Cambridge, UK), mouse anti-NF200 (1:200, Neurofilament, Sigma, Saint Louse, USA), rabbit anti CSF1 (1:100, colony-stimulating factor 1, Novus Biological), mouse anti-OX42 (1:250, AbD Serotec Inc., Raleigh, USA), mouse anti-NG2 (1:100, membrane-spanning chondroitin sulfate proteoglycan, Millipore, Merck S.p.a., Milan, Italy), mouse anti-CNPase (1:250, 2′, 3′-cyclic nucleotide 3′-phosphodiesterase, Millipore, Merck S.p.a., Milan, Italy), rabbit anti-GFAP (1:500, glial fibrillary acidic protein, Dako, Milan, Italy), and mouse anti-GFAP (1:500, Sigma, Saint Louse, USA). FluoroMyelin™ Fluorescence Myelin Staining (Molecular Probes, Eugene, OR) was also performed to stain the myelin sheaths, following the manufacturer’s specifications. After rinsing in PBS, the sections were incubated at 37 °C for 30 min with the secondary antibodies: DyLight488-conjugated affinity-pure goat anti-mouse IgG (ThermoScientific, Milano, Italy), Rhodamine Red™-X-conjugated affinity-pure donkey anti-rabbit IgG (Jackson Immunoresearch, West Grove, PA, USA), DyLight488-conjugated affinity-pure donkey anti-rabbit IgG (ThermoScientific, Milano, Italy), and Red™-X-conjugated affinity-pure donkey anti-goat IgG (Jackson Immunoresearch, West Grove, PA, USA) diluted in PBS–0.3% Triton X-100, 1:100. The sections were then rinsed in PBS and mounted in phenylenediamine solution (0.1% 1,4-phenylenediamine, 50% glycerine, carbonate/bicarbonate buffer pH 8.6, Sigma, Saint Louse, USA). The control slices were incubated with the secondary antibodies only and processed in parallel. Images were captured using a Nikon Eclipse E600 microscope equipped with digital CCD camera Q Imaging Retiga-2000RV (Q Imaging, Surrey, BC, Canada). To analyze the inflammatory infiltration, the sections were stained with toluidine blue and evaluated on three replicate sections per animal, counting the number and severity of cellular infiltrates over each whole coronal section. Cellular infiltrates were scored as follows: 0, none; 1, a few inflammatory cells; 2, organization of perivascular infiltrates; and 3, increasing severity of perivascular cuffing with extension into the adjacent tissue [28]. The inflammation score derives from the sum of infiltration scores in each cellular infiltrate.

### Spinal cord mRNA analysis

At post-immunization days 1, 5, 8, 11, and 18, five rats were sacrificed and the total LSC was dissected, snap frozen, and stored at −80 °C until used.

The total RNA was prepared from the spinal cord using QIAzol Reagent, cleaned with RNeasy Mini kit (Qiagen; Milano- Italy), and eluted in RNase Free Water, and purity and concentration were evaluated by spectrophotometry using NanoDrop ND-2000 (ThermoScientific, Milano, Italy). Complementary DNA (cDNA) synthesis was performed using RT^2^ First Strand kit following the manufacturer’s instructions. In brief, after incubation for 5 min at 42 °C with genomic DNA elimination mix in order to avoid any DNA contamination, a reverse-transcription mix of BC3, P2, RE2, and H_2_O was used and the transcription was performed in a final volume of 20 μl by heating first at 42 °C for 15 min, then at 95 °C for 5 min. Real-time PCR amplification was performed using the Stratagene Mx3005P multiplex quantitative PCR system (Agilent Technologies). The expression of genes involved in MS progression was carried out using RT^2^ SYBR Green qPCR Mastermix and the Multiple Sclerosis RT2 Profiler PCR Array (Qiagen). The genes are grouped according to the following: T cell activation/signaling, adaptive immunity, cytokine/chemokine inflammation, demyelination, and cellular stress. The raw data obtained was uploaded into GeneGlobe Data Analysis software (SABiosciences, Qiagen) for analysis. Relative quantification of messenger RNA (mRNA) expression was calculated using the comparative cycle threshold (C_T_) method and is expressed as Log2 fold change of expression. The fold change (2^(−Delta Delta Ct)) is the normalized gene expression (2^(−Delta Ct)) in the test sample divided by the normalized gene expression (2^(−Delta Ct)) in the control sample.

### CSF and plasma biomarker analysis

CSF sampling was adapted from the method of Liu and Duff [29]. Briefly, the rats were anesthetized by isoflurane inhalation (Gas Anesthesia System-21100, Ugo Basile, Varese, Italy) and placed prone on the stereotaxic instrument letting the body of the rat laid down. A sagittal incision of the skin was made below the occiput, and the subcutaneous tissue and neck muscles through the midline were separated and held apart using a microretractor. The dura mater of the cisterna magna was then penetrated by an 8-cm long glass capillary, which had a narrowed tip with an inner diameter of about 0.5 mm so that the CSF flowed into the capillary. After collection, each sample was centrifuged at 2000×*g* for 10 min at 4 °C, and the supernatant aliquoted and stored at −80 °C for biochemical assays.

Blood was collected from the abdominal aorta in EDTA-K2 Vacuntainer tubes and centrifuged at 3000×*g* for 10 min at 4 °C, and the plasma was collected, aliquoted, and stored at −80 °C until used.

Proteins known to play key roles in neuroinflammation pathways were selected. For this purpose, Bio-Plex Pro™ Rat Cytokine 24-plex Assay (Bio-Rad; Milano, Italy) was used. The kit included EPO, G-CSF (CSF3), GM-CSF (CSF2), GRO/KC, IFN-γ, IL-1α, IL-1β, IL-2, IL-4, IL-5, IL-6, IL-7, IL-10, IL-12p70, IL-13, IL-17A, IL-18, M-CSF (CSF1), MCP-1 (CCL2), MIP-1α (CCL3), MIP-3α (CCL20), RANTES (CCL5), TNF-α, and VEGF.

The simultaneous quantification of the different proteins in CSF and plasma was performed using xMAP technology and a MAGPIX Luminex platform. This technology makes use of different populations of color-coded beads of monoclonal antibodies specific to a particular protein, thus allowing simultaneous capture and detection of specific analytes from a sample. All the beads from each set are read off, which further validates the results. Using this process, xMAP Technology allows multiplexing of up to 50 unique bioassays within a single sample, both rapidly and precisely [30, 31]. In brief, after the incubation of a specific monoclonal antibody conjugated bead population with 50 μl of CSF/plasma samples for 1 h at RT, washed beads were incubated with detection antibody solution at RT for 30 min, then with the streptavidin–phycoerythrin conjugated solution (RT, 10 min). After washing, beads were resuspended in the assay buffer, shaken for 1 min and then a reading performed on the MAGPIX instrument. The results were analyzed with xPONENT 4.2 ® software and expressed as pg/ml.

### Statistical analysis

Student’s *t* test to compare means of two experimental groups, one-way ANOVA followed by Dunnett’s multiple comparison tests, and two-way ANOVA followed by Bonferroni post-test were used. Data are presented as mean ± standard error of the mean, and significance was set at *P* ≤ 0.05. All statistical analyses were performed using GraphPad Prism 6.0 (GraphPad Software).

For the normalization of gene expression on the RT^2^ PCR Profiler Array, five housekeeping genes, ribosomal protein, large, P1, hypoxanthine phosphoribosyltransferase 1, ribosomal protein L13A, lactate dehydrogenase A, and actin beta were used. The CT was determined for each sample and normalized to the average CT of the five housekeeping genes. A comparative CT method was used to calculate relative gene expression. Data are represented as Log2 fold change relative to control. The *P* values were calculated on the basis of a Student’s *t* test of the replicate 2^(–Delta Ct) values for each gene in the control group and treatment groups, and *P* values less than 0.05 was considered significant.

## Results

### Clinical profile and histopathology

**Fig. 1:**
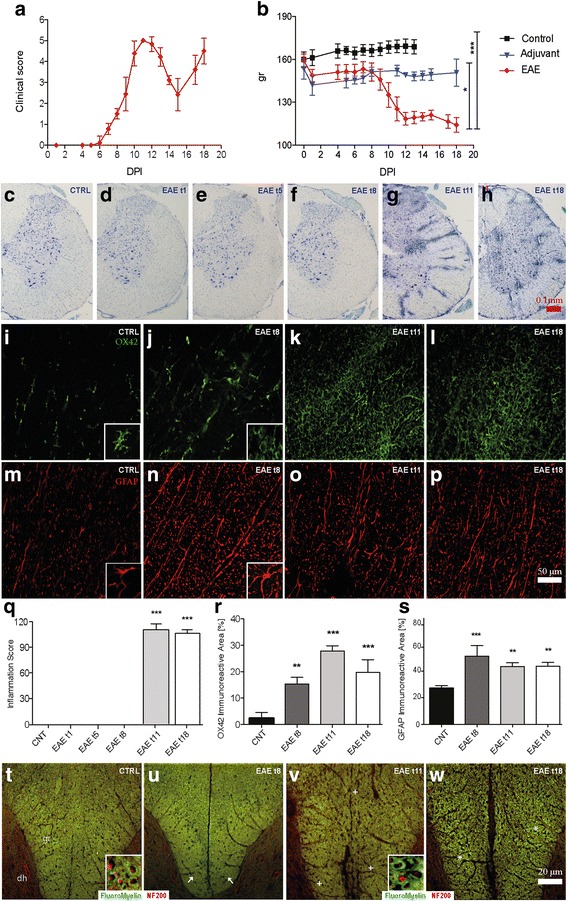
EAE clinical profile and histopathology. **a** The time-course of the neurological disability score of EAE animals is reported in the graph (mean ± SD), showing the peak at day 11 (acute phase), the remission phase at day 16, and relapse at day 18. **b** The body weight gain in experimental animals is reported in the graph (mean ± SD), revealing a significant difference between the control and EAE group. Statistical analysis: one-way ANOVA, ****P < 0.0001. Toluidine blue staining of the lumbar spinal cord (**c**–**h**) massive cellular infiltrate starting from 11 DPI (EAE t11) OX42-IR microglia staining (**i**–**l**). GFAP-immunostaining (**m**–**p**). **q** The semiquantitative evaluation of the inflammation score in the LSC. **r** Immunoreactivity indicates that microglia activation starts at 8 DPI. **s** Astrocyte immunoreactivity starts at 8 DPI. Fluoromyelin staining (**t**–**w**) in control (**t**); EAE t8 (**u**); EAE t11, the acute phase (**v**); and EAE t18, the remission-relapsing phase (**w**). *Arrow* in **u** indicate a partial demyelination at 8 DPI; *plus* in **v** indicates severe demyelination during the acute phase; *asterisks* in **w** indicate a partial recovery at 18 DPI. Statistical analysis: one-way ANOVA and Dunnett’s multiple comparison test (*P < 0.05, **P < 0.01, ***P < 0.001). *t* time

The clinical profile of EAE is reported in Fig. 1a, b in which the clinical score (a) and body weight graphs (b) are shown. Clinical signs of neurological disabilities in EAE started at 7–8 DPI and reached the higher score at 11 DPI (acute phase). A remission phase is then observed, from 12 to 15 DPI, followed by a rapid increase in the clinical score (relapsing phase). Figure 1b shows the body weight gain, which decreases in EAE groups from 2 DPI compared to the control group (*P* < 0.001). A difference between EAE and adjuvant groups is also observed from 7 to 12 DPI (*P* = 0.0139), in close correspondence with the evolution of the symptoms of the disorder, which is followed by a partial recovery.

Histopathology was performed in the LSC at each time point investigated, focusing on inflammation and demyelination. Representative images of toluidine blue staining (C-H) and immunofluorescence visualization of OX42-IR microglia cells (I-L), GFAP-IR astrocytes (M-P), and myelin (T-W) are reported in Fig. 1. Toluidine staining illustrates the massive cellular infiltrate starting from 11 DPI. The semiquantitative evaluation reveals a severe and diffuse cellular infiltrate (*P* < 0.0001) in the LSC at the acute and the remission phases (11, 18 DPI) (Fig. 1q). Significantly, microglial reaction starts at 8 DPI (*P* = 0.0001) (Fig. 1r), when OX42-IR cells take on a rounder morphology (see high-magnification inserts in Fig. 1i, j, EAE t8, compared to the control) and converge around blood vessels, reaching a peak at 11 DPI (*P* < 0.0001). The astroglial reaction was also analyzed by GFAP-immunostaining and a higher immunoreactivity was observed at 8 DPI (*P* = 0.0005) (Fig. 1m, n, control, compared to EAE t8). Demyelination signs appear at 8 DPI as revealed by the slight decrease in FluoroMyelin staining intensity in the gracile fasciculus (gr) of the LSC, (Fig. 1t, u, control, compared to EAE t8; see arrows), which is very extensive at 11 DPI (Fig. 1t, v, EAE t11, compared to the control; see plus) and has partially recovered at 18 DPI (Fig. 1t, w, control, compared to EAE t18; see asterisks).

### Regulation of inflammatory mediators in SC during early asymptomatic EAE

**Fig. 2:**
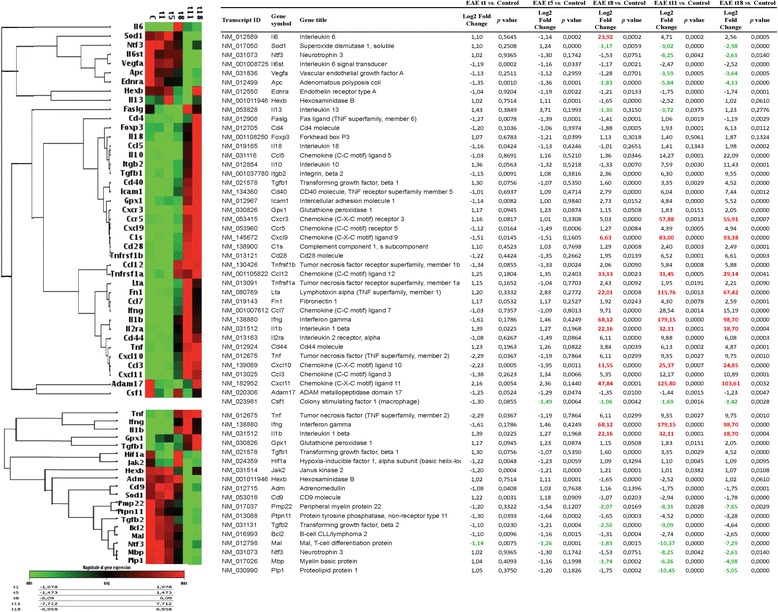
Inflammation genes mRNA expression level in the SC. The expression levels of mRNA of T cell activation/signaling, adaptive immunity, and cytokine/chemokine inflammation and demyelination are reported in the clustergram, based on heat map with dendrograms, indicating the co-regulated genes across groups. *Red color* for a gene indicates expression above the median, and *green color* indicates expression below the median. The table presents differentially expressed genes. Statistical analysis was performed using Student’s *t* test of the replicate 2^(−Delta Ct) values for each gene in the control group and treatment group; *P* < 0.05 was considered significant

The mRNA expression of genes that encode for T cell activation/signaling, adaptive immunity, cytokines/chemokines, and cellular stress involved in neuroinflammation and demyelination processes were studied in the SC by real-time PCR. The complete list of investigated genes is presented in Fig. 2. For biological averaging and variance reduction, samples from each group were pooled for microarray experiments, in fact, for very small designs, pooling dramatically improves accuracy [32–34]. The results from the different groups are presented in a clustergram that performs non-supervised hierarchical clustering to display a heat map with dendrograms indicating co-regulated genes across groups, criteria for significance are reported in the table of magnitude gene expression (Fig. 2). Several pro-inflammatory cytokines and chemokines such as IL-1β and CCL12 were highly up-regulated in the SC at 8 DPI and reached a peak at 11 DPI. IFN-γ, CXCL11, and LTA were the most up-regulated genes, showing more than 22 Log2 fold change compared to the controls at 8 DPI, around 115 at 11 DPI, and more than 67 Log2 fold change at 18 DPI. The highest up-regulation was observed for IFN-γ, 68.12 Log2 fold change at 8 DPI, 179.15 at 11 DPI, and 98.7 at 18 DPI (Fig. 2). Notably, ADAM17 and CSF1 mRNAs are strongly down-regulated at 1 DPI. Most of the anti-inflammatory cytokines and chemokines were down-regulated with a profile opposite to that observed for the pro-inflammatory ones (starting to decrease from 8 DPI), including VEGF, SOD1, NTF3, APC, HEXB, while an up-regulation was observed for some anti-inflammatory mediators such as GPX1 and IL-10. With regard to genes involved in myelination, a down-regulation starting from 8 DPI was observed, including MAL, MBP and PMP22. To note, the overexpression of IFN-γ mRNA in EAE in acute phase (11 DPI) is the highest EAE-induced up-regulation observed compared to the other inflammatory mediators investigated (Fig. 2).

### Inflammation biomarkers at plasma and CSF level are regulated during early asymptomatic EAE

**Fig. 3:**
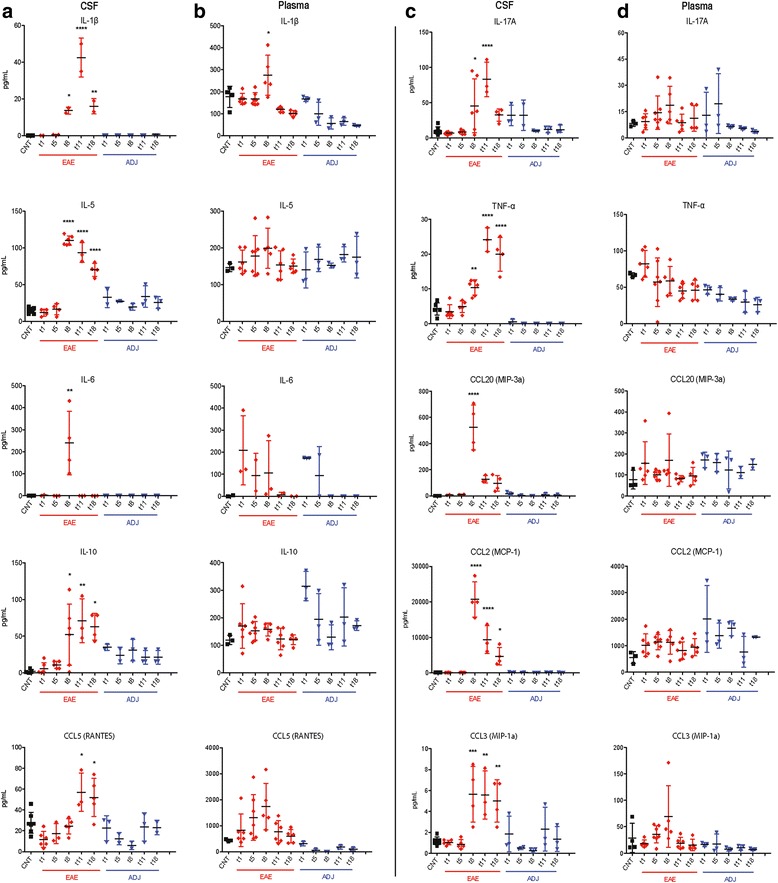
Cytokine/chemokine levels in CSF/plasma. The amount of IL-1b, IL-17A, IL-5, TNF-α, IL-6, MIP-3a (CCL20), IL-10, MCP-1 (CCL2), RANTES (CCL5), and MIP-1a (CCL3) in CSF (**a**, **c**) and plasma (**b**, **d**) in the control, EAE, and adjuvant groups are reported. Results are presented as individual values (pg/ml), and the mean ± SD is also shown. Statistical analysis: one-way ANOVA and Dunnett’s multiple comparison test (*P < 0.05, **P < 0.01, ***P < 0.001, ****P < 0.0001)

In order to evaluate the kinetics of inflammation biomarkers during EAE, 24 cytokines and chemokines, EPO, G-CSF (CSF3), GM-CSF (CSF2), GRO/KC, IFN-γ, IL-1α, IL-1β, IL-2, IL-4, IL-5, IL-6, IL-7, IL-10, IL-12p70, IL-13, IL-17A, IL-18, M-CSF (CSF1), MCP-1 (CCL2), MIP-1α (CCL3), MIP-3α (CCL20), RANTES (CCL5), TNF-α, and VEGF, were simultaneously quantified in CSF samples at the different time points investigated (1, 5, 8, 11, and 18 DPI, Fig. 3a, c). IL-17A, IL-1β, TNF-α, and IL-10 were significantly increased at 8 DPI in EAE group compared to the control animals (*P* = 0.0412; *P* = 0.0127; *P* = 0.0039; *P* = 0.0412, respectively), reaching a peak at 11 DPI (*P* < 0.0001; *P* < 0.0001; *P* < 0.0001; *P* = 0.0074, respectively) and decreasing again at 18 DPI (ns; *P* = 0.0035; *P* < 0.0001; *P* = 0.0128, respectively). For both pro-inflammatory chemokines CCL2 and CCL3 and for the anti-inflammatory cytokines IL-5, the same significant profile was observed, reaching a peak at 8 DPI (*P* < 0.0001; *P* = 0.001; *P* < 0.0001, respectively) then starting to decrease starting from 11 DPI (*P* < 0.0001; *P* = 0.0046; *P* < 0.0001, respectively). The levels of CCL20 and IL-6 were significantly increased in EAE animals only at 8 DPI (*P* < 0.0001; *P* = 0.004, respectively) (Fig. 3). Unpredictably, IFN-γ, IL-13, IL-2, IL-1α, IL-4, IL-12p70, and GM-CSF were not detected at any of the time points analyzed in the CSF in our experimental conditions.

The different cytokines/chemokines quantified in CSF were also simultaneously measured in plasma samples (Fig. 3b, d) in order to both correlate with CSF changes and to evaluate the peripheral effect of adjuvant. IL-1β increased in EAE compared to control group of animals at 8 DPI (*P* = 0.0231). GM-CSF was the only cytokine not detectable at any of the time points analyzed, and no significant changes was observed for the rest of the panel compared to the control.

No significant variations between adjuvant and control groups were observed in CSF or plasma at the investigated times.

### Immunohistochemical analysis

**Fig. 4:**
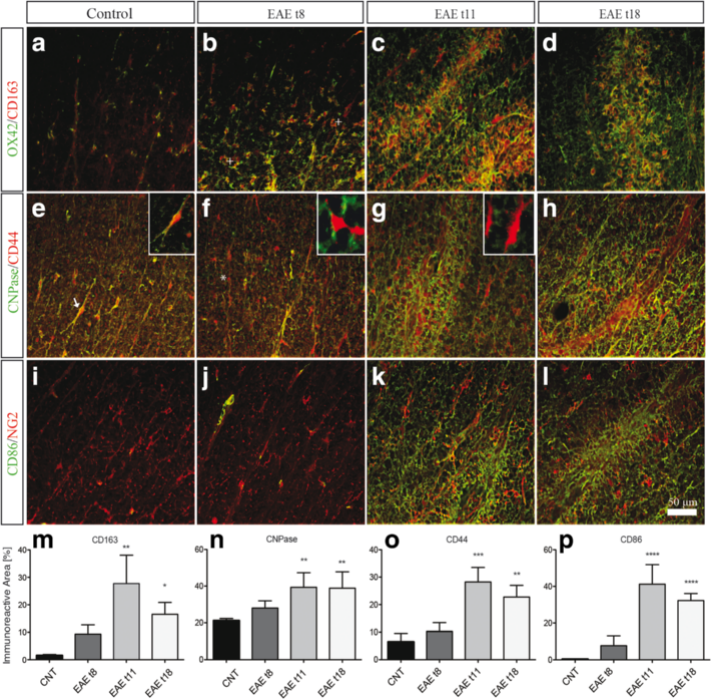
Immunohistochemistry analysis. Double immunostaining of OX42/CD163, CNPase/CD44 and CD86/NG2 in the lumbar spinal cord of control and EAE rats*.* **a**–**d** Double labeling for microglial cells marker (OX42) and M2 macrophages marker (CD163) in the white matter of control (**a**) and EAE experimental groups: 8, 11, and 18 DPI (**b**–**d**). *Plus* in **b** indicates the activated microglia expressing CD163. **e**–**h** Double labeling for oligodendrocyte marker (CNPase) and T lymphocyte marker (CD44) (see also the included high-power magnifications). CD44 is expressed by oligodendrocytes in control. *Arrows* in **e** indicate co-localization, although this expression changed at 8 DPI in **f. i**–**l** Double labeling for oligodendrocyte precursor cells (NG2) and M1 macrophages marker (CD86). The immunoreactive area of CD163 (**m**), CNPase (**n**), CD44 (**o**), and CD86 (**p**) in EAE rats compared to controls. Statistical analysis: one-way ANOVA and Dunnett’s multiple comparison test (*P < 0.05, **P < 0.01, ***P < 0.001, ****P < 0.0001)

In order to elucidate the cell type producing the most regulated biomarkers, double-labeling experiments were performed in the LSC of the control and EAE animals. Representative images from the control and EAE animals at 8, 11, and 18 DPI are shown in Fig. 4. The following markers were investigated: OX42 as marker for microglia; CD86, as marker for M1 macrophages phenotype; CD163, as marker for M2 macrophages phenotype; CD44, as marker for T lymphocytes; NG2, as marker for oligodendrocyte precursor cells (OPCs); and CNPase, as marker for oligodendrocytes. Immunostaining was quantified by a computerized procedure to evaluate the immunoreactive area. As expected, the increase in OX42-IR observed at 8 DPI corresponds to increased expression of the M2 cytokine CD163 (Fig. 4a–d, see plus in B, M), while the M1 macrophage marker CD86 showed the highest immunoreactive area at 11 DPI (Fig. 4i–l, p). This activation was accompanied by an increase in the CNPase immunoreactive (IR) area starting from 8 DPI (Fig. 4e–h, n), which partially contrasts with the FluoroMyelin staining results (Fig. 1t–w). In the control group, CD44 was expressed by oligodendrocytes (Fig. 4e; see arrows), while this co-localization was no longer present in the EAE animals at 8, 11, and 18 DPI (Fig. 4f; see asterisk). The highest up-regulation of the different markers verified in the white matter was detected in the acute phase of EAE (Fig. 4c, g, and k), where also a significant up-regulation of CD163 (*P* = 0.0016), CNPase (*P* = 0.0197), CD44 (*P* = 0.0005), and CD86-IR (*P* < 0.0001) areas were observed at 11 DPI (Fig. 4m, n, o, and p, respectively).

No significant results were observed in EAE t1, EAE t5, and adjuvant experimental groups (results not shown).

### CSF1 regulation in early presymptomatic EAE

**Fig. 5:**
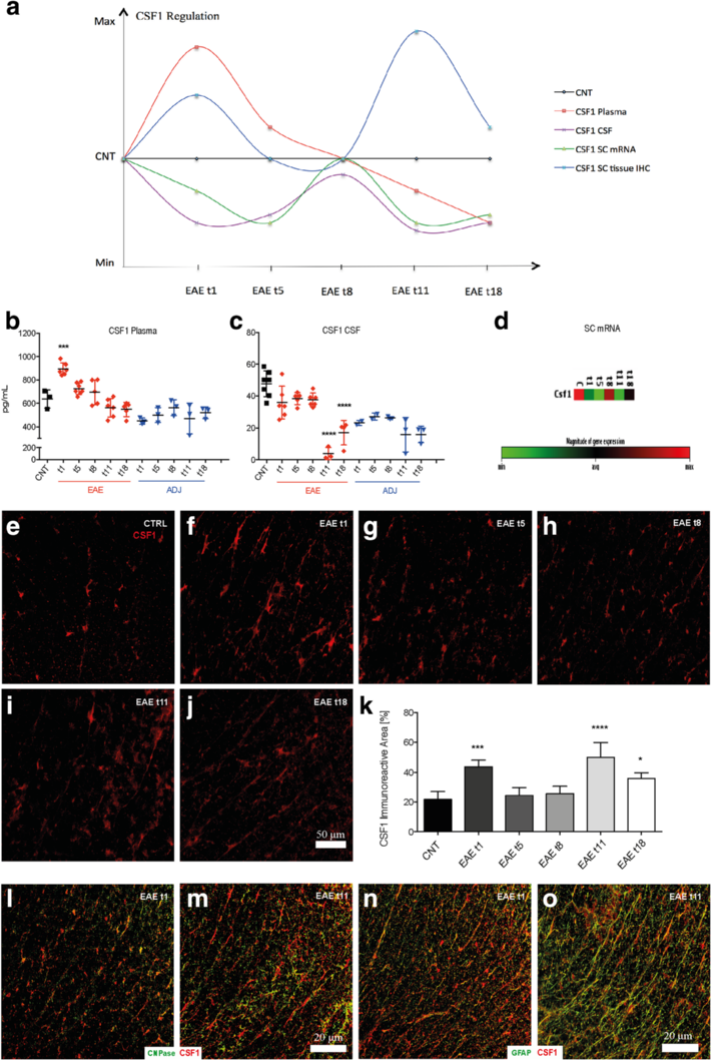
Early regulation of CSF1 in EAE. **a** Schematic illustration of CSF1 regulation in all the biological samples analyzed on Excel: the XY scatter option is chosen and under the chart subtypes, the option connecting the points with *smooth lines* with markers is selected. The amount of CSF1 in plasma (**b**) and CSF (**c**) in the control, EAE, and adjuvant groups are reported. Results are presented as individual values (pg/ml), and the mean ± SD is also shown. **d** The expression level of mRNA of CSF1 is reported in the clustergram, based on heat map with dendrograms, indicating the co-regulation across the different groups (**e**–**j**) **k** CSF1-immunostaining in the white matter of control and EAE experimental groups: 1, 5, 8, 11, and 18 DPI. The immunoreactive area of CSF1 in EAE rats compared to controls. Double labeling for CSF1 and oligodendrocytes marker (CNPase) and GFAP in the EAE group 1 and 11 DPI (**l**, **m** and **n**, **o**, consecutively). Statistical analysis: one-way ANOVA and Dunnett’s multiple comparison test (*P < 0.05, ***P < 0.001, ****P < 0.0001)

**Fig. 6:**
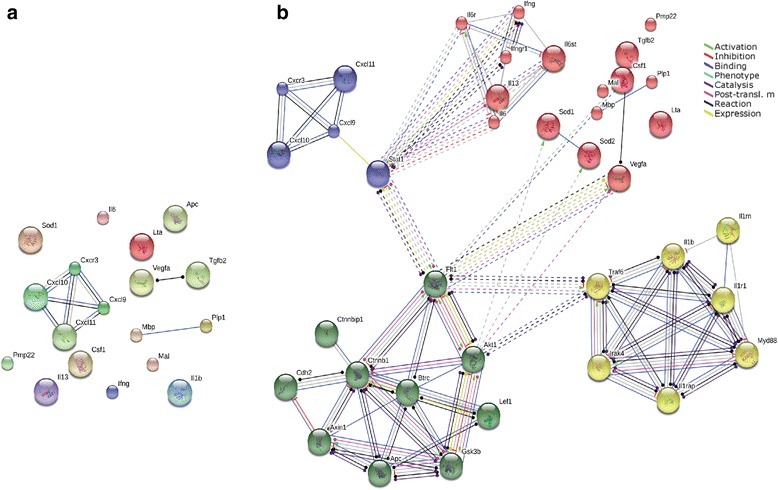
Functional pathway and network analysis. **a** Illustration of protein interactions of the most regulated gene at 8 DPI upon querying the STRING 10.0 protein network (evidence view). **b** The extended net (high confidence, 0.7)*. Nodes* represent proteins; *same node colors* indicate membership of the same cluster, and *different line colors* denote the type of evidence for the interaction

Going one step deeper into biomarker regulation at the early phase of EAE, it was found that the pro-inflammatory mediator CSF1 (The CSF-1R is a member of the platelet-derived growth factor receptor (PDGFR) family of class III receptor tyrosine kinases that includes PDGFRα/β, stem cell factor receptor (c-Kit), and Flt3/Flk2) [35] was regulated starting from 1 DPI in EAE group compared to the control in all the biological samples analyzed (Fig. 5a). In plasma, CSF1 was significantly up-regulated only at 1 DPI (*P* = 0.0006), then continuously decreased till the last time point studied, 18 DPI (Fig. 6b), while in CSF, CSF1 level started to decrease at 1 DPI, becoming significant at 11 and 18 DPI (*P* < 0.0001, *P* < 0.0001) (Fig. 5c). Notably, CSF1-IR in the tissue also increased starting from 1 DPI (*P* = 0.0003) (Fig. 5e, k), then decreased at 5 and 8 DPI (Fig. 5g, h) ultimately reaching the highest immunoreactivity at 11 DPI (*P* < 0.0001) (Fig. 5i, k). The mRNA expression of CSF1 gene in SC was also analyzed (Fig. 2), and a down-regulation at 1 and 11 DPI was observed (Fig. 5d). In the attempt to identify the CSF1-producing cells at 1 and 11 DPI, double-labeling immunostaining of CSF1 with CNPase and GFAP was performed in the LSC (Fig. 5l–o). Figure 6n, o shows that CSF1 is constitutively expressed by astrocytes not only in the EAE animals but also in the control (results not shown). Interestingly, CSF1 was expressed by oligodendrocytes at 1 DPI in EAE (Fig. 5l), while this expression is no longer present as from 11 DPI (Fig. 5m).

### Functional pathway and network analysis

In an exploratory study, a pathway analysis approach of the different proteins derived from the most early regulated genes at 8 DPI was implemented. This time point corresponds to the beginning of the regulation of genes in the CNS, while cellular infiltrates are not yet present and clinical signs start to appear. We proceed through the web-software STRING 10.0 (http://string-db.org/) and Gene Ontology databases. The protein–protein interaction analysis (both physical and functional interactions) was performed using default parameters (high confidence, 0.7) and R.norvegicus as the organism of interest. The software allows the net of interactions including other proteins closely linked to the one analyzed to be extended, in order to obtain a better understanding of the possible pathways affected by EAE in DA rats at early onset. The analysis showed that almost all the proteins deriving from genes of interest (except for the CXC family) did not directly interact with each other (Fig. 6a). The extended network (indirect protein–protein interactions) showed that those proteins were connected in four clusters according to their involvement in the biological process (autoimmune disease), situating STAT1 (signal transducers and activators of transcription), FLT1 (vascular endothelial growth factor receptor 1), and TRAF6 (TNF receptor associated factor) as nodes of the extended net linking the different groups (Fig. 6b). To gain further insight into the biological significance of those clusters in MS, an enrichment analysis using Gene Ontology was performed. It was found that these four clusters consist of a Wnt signaling pathway (the green cluster), a cytokine-mediated signaling pathway (the yellow cluster), T cell chemotaxis (the blue cluster), and positive regulation of mitogen-activated protein tyrosine kinase (MAPK) (the red cluster). Notably, CSF1 takes part of MAPK cluster.

### Effects of selective inhibition of CSF1R activity on the clinical score of EAE animals

**Fig. 7:**
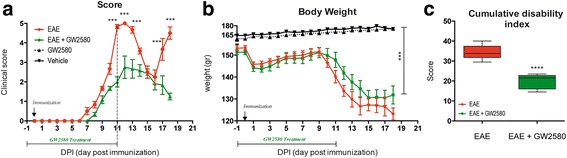
Effects of selective inhibition of CSF1R activity on the disease progression. **a**. Time-course of the neurological disability score of EAE and EAE + GW2580 animals is reported in the graph (mean ± SD), showing the peak at day 11 (acute phase), the remission phase at day 16, and relapse at day 18 for EAE animals while a delayed and reduced clinical score was observed for EAE + GW2580 group. **b** The body weight gain (mean ± SD) is reported in the graph (mean ± SD), enlightening a significant difference between GW2580 and EAE + GW2580 group. **c** Cumulative disability index. Statistical analysis: **a**, **b** two-way ANOVA and Bonferroni post-test (***P < 0.001); **c** one-way ANOVA, ****P < 0.0001

Since the peculiar temporal expression pattern of CSF1 in particular in the very early phase of the disease, the tyrosine kinase activity of CSF1R was inhibited by the oral administration of GW2580. GW2580 is a highly selective inhibitor of the c-FMS kinase, and through this pathway, this small molecule blocks CSF1 signaling. Treatment started 1 day before the immunization and till 11 DPI. The clinical profiles of the disease in EAE, EAE + GW2580, control + GW2580, and vehicle groups of animals are reported in Fig. 7, in which the clinical score (a) and body weight graphs (b) are shown. Clinical signs of the neurological disabilities in EAE were the same as reported in the first group of animals included in our study (Fig. 1a), which prove the reproducibility of the model. While the clinical signs started at 6–7 DPI in the EAE group, a delay in the appearance of the neurological disability was observed in the EAE + GW2580 group. The acute phase was at 11–12 DPI with a maximum score of 5, while a significant reduction of the severity of the disease was observed in the EAE + GW2580 group (*P* < 0.001) with a maximum score of 2.8. Interestingly, the EAE + GW2580 animals did not show any relapse phase (*P* < 0.001) and after the acute phase, they were recovering till the 18 DPI, last day of the experiment (*P* < 0.001). Figure 7b shows the body weight gain. A decrease was observed in the EAE + GW2580 groups from 2 DPI compared to the GW2580 animals (*P* < 0.001), which is the same profile of the body weight loss observed in the EAE group but less severe, in close correspondence with the evolution of the symptoms of the disorder, which is followed by a recovery. We also calculated the cumulative disability index, reported in Fig. 7c, observed in EAE compared to the EAE + GW2580 groups (*P* < 0.001).

## Discussion

The aim of this study was to investigate the expression profile of molecules involved in inflammation and demyelination in the very early presymptomatic phase of EAE induced by active immunization in female DA rats by comparing the tissue (spinal cord), CSF, and plasma. It was previously demonstrated that in the acute phase of this model, a massive infiltration of inflammatory cells and extensive demyelination were observed in the spinal cord [36–38]. Samples collected at symptom-free times (1 and 5 DPI) and at the symptom onset (8 DPI), peak (11 DPI), and relapse (18 DPI) were then examined using high-throughput technologies together with bioinformatic analysis for molecule and pathway identification in CNS tissue and biological fluids.

At mRNA level (spinal cord tissue), T cell activation/signaling, adaptive immunity, cytokine/chemokine inflammation, demyelination, and cellular stress were analyzed, thereby including astroglial and microglial markers. Microglia markers refer to the “resting state” (M0); to inflammatory microglia (M1 polarization) producing numerous pro-inflammatory cytokines/chemokines; and to anti-inflammatory microglia (M2 polarization), which triggers them to endorse regeneration and eliminate debris [39–41]. The most significant results were verified by means of double-labeling immunohistochemistry. At protein level in CSF and plasma, 24 cytokines were analyzed.

Our first important result is that an early regulation of most of the inflammatory and demyelinating biomarkers was observed as soon as symptoms appear (8 DPI) but before the disease peak (11 DPI). Molecular markers of microglial activation toward a phagocytic phenotype are found (8 DPI), when also astrocyte hypertrophy is observed. The highest regulation of these markers is observed when symptoms peak (11 DPI, acute phase) and then partially recover at relapse (18 DPI). Significantly, microglia and astrocytes were activated early in the EAE animals by 8 DPI. Indeed, almost all the cytokines/chemokines shift their expression at 8 DPI in plasma, CSF, and even in the SC. In particular, a higher regulation of CCL2, TNF-α, and IL-1β was found, possibly indicating the initiation of BBB breakdown. Indeed, it has been demonstrated that the CCL2-CNS level can be induced by the pro-inflammatory cytokine IFN-γ, TNF-α, and IL-1β and does affect the BBB integrity and attract monocytes to the CNS [42, 43]. Interestingly, we found that the anti-inflammatory biomarkers, IL-5 and IL10, have the same profile of expression as the pro-inflammatory mediators, revealing that both “M1” and “M2” polarization is present at the same time during EAE. Actually, very recent studies have shown that microglia/macrophages simultaneously express both “M1” and “M2” phenotypic markers in brain trauma, suggesting that these cells display a mixed phenotype due to the fact they cannot adequately switch to a polarized “M1-only” or “M2-only” phenotype [44, 45].

Using a functional pathway analysis tool, Gene Ontology and Enrichment Analysis, four possible pathways were indicated as affording suggestive evidence of being associated with the early phase of MS: the Wnt signaling pathway, the cytokine-mediated signaling pathway, the T cell chemotaxis pathway, and MAPK. Significantly, Wnt signaling has been recognized as the central tenet in the development and regeneration of myelin in the CNS [46]. Chronic activation of canonical Wnt signaling in oligodendrocyte precursors results in delayed developmental and regenerative myelination [47, 48]. Interestingly, IL-6, which belongs to the MAPK pathway, was detected only at 8 DPI. Besides, IL-17A, which is secreted by T helper-17 which contributes to pathogenesis in MS and EAE [49], started to be regulated at 8 DPI, confirming that IL-6 promotes T cell development at 8 DPI. Indeed, it has been shown that IL-6-deficient mice were resistant to EAE because they fail to induce CNS–T helper-17 cells [50].

Our second major result is that the colony-stimulating factor CSF1, part also of MAPK pathway, was regulated at 1 DPI in EAE groups compared to the control, not only in the plasma but also in CSF and SC. CSF1, is an integral tyrosine kinase transmembrane receptor and signals through its receptor CSF1R (also known as c-FMS) to regulate the differentiation, proliferation, and recruitment of microglia [51, 52]. Thus, our data indicate that the first sign of immunological response in EAE occurs in the CNS already 24 h after immunization and plasma biomarker could reflect microglia activation in the tissue. It was then attempted to identify the cell type producing CSF1. It was found that CSF1 at 1 DPI is expressed by oligodendrocytes and astrocytes in the EAE animals, while in the acute phase (11 DPI), only astrocytes express CSF1. However, it was also seen that CSF1 is expressed by GFAP-positive cells in control, adjuvant-injected, and intact animal. Moreover, we found that the treatment with GW2580, a selective inhibitor of CSF1R, delayed the onset of the disease, significantly reduced the clinical severity and surprisingly prevented the relapse phase even though the treatment was performed till the 11 DPI, thus suggesting the CSF1 is a major molecular regulator in the very early phases of EAE. To our knowledge, our results provide the first evidence of the very early regulation of CSF1 in EAE and on a major role of CSF1-mediated events in EAE onset and progression.

A major question arising from this result is the cell type responsible for this very early effect. The CSF1 receptor (CSF1R), encoded by the proto-oncogene *c-fms* [53, 54] and having CSF1 and IL-34 as natural ligands [55], is expressed by several cell types, including macrophages and microglia [56]. In the brain, it has been confirmed that under normal conditions, microglia are the only cell type that expresses the CSF1R [57, 58]. Several lines of evidence suggest that microglial activation is mediated by the binding of CSF1 to CSF1R, which triggers the release of inflammatory cytokines [59, 60]. A role of CSF1 in microglial activation and disease progression has been described in several models of injury and neurodegenerative diseases, but in few of them, the responsible cell type has been identified. In neuropathic pain induced by peripheral nerve injury, CSF1 is produced and retrograde transported to the spinal cord by sensory neurons [61]. In the mouse model of amyotrophic lateral sclerosis, the positive effects of GW2580 has been attributed to the attenuation of macrophages infiltration of the peripheral nerve [62]. In an Alzheimer’s mouse model, prolonged treatment with GW2580 directly target microglia regulating cytokines production and also improving the functional outcome [25]. When used in EAE starting from 10 DPI, GW2580 reduces the proportion of peripheral macrophages, also decreasing the number of inflammatory foci in the CNS [63]. This effect has at least partially attributed to the repression of the autocrine signaling of inflammatory macrophages and microglia, leading to the reduction of neuroinflammatory cytokines and chemokines [57, 64, 65], suggesting that targeting tyrosine kinase receptors such as c-Fms and PDGFR could prevent the development of the disease by enhancing BBB integrity [66].

According to our results, it could thus be speculated that CSF1-CSF1R signaling plays an important role in not only the early phase of EAE but also all along the disease progression. CSF1 could be so a good trigger for microglial activation and subsequent induction of neuroinflammation, and its increase could be detected in biological fluids including plasma. Further studies will elucidate the mechanism of this early microglial activation, also in view of possible therapeutic implications. In fact, the therapeutic potential of the CSF1R kinase inhibitor has been explored in many pathologies such as cancer, bone disease, inflammatory diseases, and other autoimmune disorders, and so several CSF1R tyrosine kinase inhibitors are under development for clinical applications [67–70].

## Conclusions

In the attempt to elucidate still elusive aspects of multiple sclerosis (MS) pathogenesis, we performed a time-course study in experimental allergic encephalomyelitis (EAE), the most widely used MS rodent model, focusing on the presymptomatic phase. By investigating neuroinflammation and demyelination biomarkers in the tissue, CSF, and plasma using high-throughput technology and bioinformatics analysis, we discovered the very early regulation of the chemokine colony-stimulating factor 1 (CSF1), which indicates the occurrence of microglia activation already 1 day after immunization. The selective inhibition of CSF1R decreased EAE clinical severity and prevents the relapse phase suggesting the importance of CSF1-CSF1R signaling in microgliosis and inflammation in MS.

## Abbreviations

BBB: Blood–brain barrier
CCL: C-C-L motif chemokine
CNS: Central nervous system
CSF: Cerebrospinal fluid
CSF1: Colony-stimulating factor 1
CXCL: C-X-C motif chemokine
DPI: Day post-immunization
DA: Dark agouti
EAE: Experimental allergic encephalomyelitis
GFAP: Glial fibrillary acidic protein
IC: Inhibitory concentration
IFN-γ: Interferon-γ
IL: Interleukin
IR: Immunoreactivity
LSC: Lumbar spinal cord
MAPK: Mitogen-activated protein tyrosine kinase
MS: Multiple sclerosis
SC: Spinal cord
TNF: Tumor necrosis factor
